# Design and Validation of Single-Axis 3D-Printed Force Sensor Based on Three Nested Flexible Rings

**DOI:** 10.3390/s24165441

**Published:** 2024-08-22

**Authors:** Pengfei Yang, Shiwei Xin, Yuqing Mao, Fei Dang, Feng Huang

**Affiliations:** 1 School of Mechanical Engineering and Automation, Fuzhou University, Fuzhou 350108, China; pengfeiyang@fzu.edu.cn (P.Y.); shiweixin2022@126.com (S.X.); yuqing_mao@126.com (Y.M.); 2 College of Physics and Electronic Information Engineering, Minjiang University, Fuzhou 350108, China

**Keywords:** 3D-printed force sensor, nested rings, force-displacement relationship, nonlinear analytical model

## Abstract

Force measurement is crucial in numerous engineering applications, while traditional force sensors often face problems such as elevated expenses or significant measurement errors. To tackle this issue, we propose an innovative force sensor employing three nested flexible rings fabricated through 3D additive manufacturing, which detects external forces through the displacement variations of flexible rings. An analytical model on the basis of the minimal energy method is developed to elucidate the force-displacement correlation with nonlinearity. Both FEM simulations and experiments verify the sensor’s effectiveness. This sensor has the advantages of low expenses and easy manufacture, indicating promising prospects in a range of applications, including robotics, the automotive industry, and iatrical equipment.

## 1. Introduction

As a vital part of modern science and engineering, the force sensor (FS) is widely used in industrial automation, medical diagnostics, and sports biomechanics [1,2,3,4]. Due to the inherent difficulty in directly measuring force, FSs employ various fundamental principles to convert external forces into measurable physical quantities [5]. Consequently, numerous types of FSs have been developed and reported in the literature, including the displacement FS [6,7,8], the capacitance FS [9], the resistance FS [10,11], the strain FS [12,13], the voltage FS [14,15], the magnetic induction FS [16,17], and the optical FS [18]. Despite their extensive utilization and evolution, some traditional FSs often experience significant measurement errors [19,20,21,22,23,24,25,26,27,28,29]. In contrast, some traditional FSs with minimal measurement errors often possess high expenses [30], posing challenges in meeting the request for precise and cost-effective force-sensing solutions. For instance, commercial FSs based on the strain gauge with minimal measurement errors typically utilize premium-quality fabrication materials, such as highly purified metals, and buying and manufacturing such materials cause significant costs, thereby increasing the cost and mass of the FSs. In this work, the FS’s measurement error denotes the maximal error percentage between the calculated force output value and the real force value throughout the sensing range [31].

Due to the rapid advancement in the 3D additive manufacturing technique in recent years, 3D-printed FSs [31,32,33,34] garner significant interest for their cost-effective, efficient fabrication process, as well as their ability to utilize various printing materials. These materials, characterized by diverse properties, facilitate the manufacture of FSs with diverse geometric dimensions and customized configurations. However, traditional 3D-printed FSs often suffer from significant measurement errors. As an instance, the new FS made by 3D additive manufacturing utilized elastic elements arranged orthogonally and exhibited an error of 2.18% across a 0–2.8 N range [31]. Similarly, the 3D-printed miniature FS showed an error of 1.14% across a 0–2.5 mN range [32]. The 3D additive manufacturing FS utilizing optical fiber grating demonstrated an error of 0.98% across a 0.9–2.7 N range [33]. The strain gauge FS fabricated by 3D additive manufacturing had an error of 3.15% across a 0–160 mN range [34]. The significant measurement error observed in conventional FSs primarily arises from nonlinear force behaviors [31,32,33,34,35]. Addressing this issue is challenging due to the difficulty in developing a precise nonlinear analytical model to characterize the nonlinear behaviors precisely. Our previous work reported the single-axis FS along with an analytical model on the basis of a 3D-printed flexible ring, which had an error of 0.5% and a range of 0–108 N [5]. However, the previous FS’s geometry was large (238 mm ∗ 240 mm ∗ 60 mm), which required a miniaturization study to decrease the sensor geometry and maintain the measurement range.

To address the aforementioned issue, we present a cost-effective FS utilizing the nested structure of three 3D-printed flexible rings complemented by a nonlinear analytical model. Subject to the external loading force, three flexible rings transform the loading force into three rings’ displacement, allowing for the applied force determination through measurement of the rings’ top displacement. The structure of this paper is as follows. Section 2 provides a detailed exposition of the sensor’s structure and measurement principle, along with the analytical analysis of the nonlinear force-displacement correlation based on the minimal energy method. Then FEM simulations are utilized to simulate the nonlinear force-displacement correlation along with the comparison with analytical results in Section 3. Section 4 presents experimental results and compares them with theoretical outcomes. Finally, Section 5 discusses the advantages of the proposed FS relative to traditional counterparts.

## 2. Structural Design and Sensing Scheme

### 2.1. Structural Design

**Figure 1:**
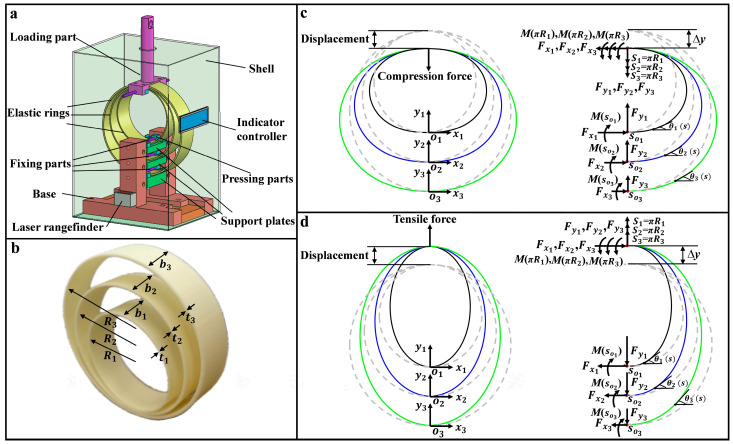
Diagram for (**a**) FS and (**b**) flexible rings. Diagrams of three rings subjected to (**c**) the *F*_compression_ and (**d**) *F*_tensile_.

The diagram illustrating the designed FS utilizing three nested flexible rings is depicted in Figure 1a. The FS comprises three distinct flexible rings, the fixing parts, the base, the laser ranger, support plates, pressing parts, the indication controller, and the loading part. The bottom of the flexible rings is stably anchored at the center of the support plates using the fixing and pressing parts. The loading part is mounted on top of the flexible rings to bond three distinct, flexible rings together and moves vertically to make certain that the three rings are loaded vertically. A laser ranger with an upward sensing orientation is positioned on the base side to obtain the height variation of three flexible rings. The laser ranger, electronically linked with the indicator controller, supplies displacement values so that the indicator controller calculates the loading force based on the force-displacement correlation of the rings.

The nested configuration of three distinct rings is pivotal in this sensor’s design. The following sections provide a detailed description of the sensor structure, operational principles, and the critical role of flexible rings in achieving force measurement. Take into account three lightweight, flexible rings that are non-stretchable, with radii *R*_1_, *R*_2_, *R*_3_, widths *b*_1_, *b*_2_, *b*_3_, and thicknesses *t*_1_, *t*_2_, *t*_3_ (*t*_1_ << *R*_1_, *t*_2_ << *R*_2_, *t*_3_ << *R*_3_), respectively, where the gravity has negligible effects on the rings [36,37], as depicted in Figure 1b. Due to the small thickness of flexible rings, they experience substantial nonlinear bending deformation when subjected to the external tensile force (*F*_tensile_) or the external compression force (*F*_compression_). Bottoms of three rings are anchored at the origins of the Cartesian coordinate systems, while their tops are subjected to either the compressive force (Figure 1c) or the tensile force (Figure 1d) in the *y*-axis. The original rounded shape of the three rings would transform into different balance configurations due to the loading of force. In compression, internal forces within three rings lead to a downward shrinkage, resulting in a height decrease in the rings. Conversely, under the tensile force, different internal forces cause the upward extension of the rings, thereby increasing their height. Since the variation in rings’ height correlates directly with the external loading force, the loading force is determined by detecting the rings’ height variation. This demands the development of a theory that delineates the force-displacement correlation of the rings.

### 2.2. Analytical Model

Considering the analogous theoretical analysis of flexible rings to either the *F*_tensile_ or the *F*_compression_, the compression force is taken as the example for the theoretical analysis, as depicted in Figure 1c. Given the symmetry of the flexible rings about the *y*-axis, we analyze the configuration of the right half for simplicity. Without external forces, no internal forces or torques are acting within the rings. With the external compression force $F_{compression}$ = $F_{y_{1}}$ + $F_{y_{2}}$ + $F_{y_{3}}$ loading at the top of the rings, internal forces and torques are generated by the rings to counteract the external compression force, as depicted in Figure 1c. The rings undergo a reduction in height until they achieve different balance configurations. The energy functional for the right half rings is formulated as follows:

(1) $$\begin{array}{l} W=\sum_{i=1}^{3}\left(\underset{\mathrm{Bending}\;\mathrm{energy}}{\underset{︸}{\frac{K_{i}}{2}\int_{0}^{\pi R_{i}}{\left(\theta_{i}^{\prime }\left(s_{i}\right)\right)}^{2}ds_{i}}}-\underset{\mathrm{Constraint}\;}{\underset{︸}{F_{x_{i}}\int_{0}^{\pi R_{i}}\cos\theta_{i}\left(s_{i}\right)ds_{i}}}-\underset{\mathrm{Work}}{\underset{︸}{F_{y_{i}}\left(\int_{0}^{\pi R_{i}}\sin\theta_{i}\left(s_{i}\right)ds_{i}-2R_{i}\right)}}\right) \end{array}$$

where *i* = 1, 2, 3 represents three rings. The bending rigidity $K_{i}=EI_{i}$ (*i* = 1, 2, 3) are related to the bending modulus *E* and the inertia moment $I_{1}$, $I_{2}$, and $I_{3}$, respectively. *s*_1_, *s*_2_, and *s*_3_ denote the arc lengths of three flexible rings. Take the bottoms of three rings as origins $s_{o_{1}}$(*s*_1_ = 0), $s_{o_{2}}$ (*s*_2_ = 0), and $s_{o_{3}}$(*s*_3_ = 0) for natural coordinates. $\theta_{1}$ (*s*_1_), $\theta_{2}$ (*s*_2_), and $\theta_{3}$ (*s*_3_) denote the deflection angle of any point on each ring relative to the *x*-axis, respectively. $\theta_{i}^{\prime }(s_{i})$ = d$\theta_{i}$/d$s_{i}$ (*i* = 1, 2, 3) represent curvature. $F_{x_{1}}$, $F_{x_{2}},$ and $F_{x_{3}}$ denote the internal forces along the *x* direction while $F_{y_{1}}$, $F_{y_{2}}$, and $F_{y_{3}}$ represent three component forces of the external compression force loaded at the rings’ top. *M* ($s_{o_{i}}$) = $K_{i}\theta_{i}^{\prime }$ ($s_{o_{i}}$) (*i* = 1, 2, 3) denote the internal torques at the bottom of the rings, while *M*(π$R_{i}$) = $K_{i}\theta_{i}^{\prime }$ (π$R_{i}$) (*i* = 1, 2, 3) represent those at the rings’ top. The constraints in the energy function present the *y*-axis symmetry of the flexible rings. Then Equation (1) is reformulated as follows:

(2) $$W=\sum_{i=1}^{3}\left(\int_{0}^{\pi R_{i}}w_{i}ds_{i}+2R_{i}F_{y_{i}}\right)$$

where $w_{i}=\frac{K_{i}}{2}(\theta_{i}^{\prime }\left(s_{i}\right){)}^{2}-F_{x_{i}}\cos\theta_{i}\left(s_{i}\right)-F_{y_{i}}\sin\theta_{i}\left(s_{i}\right)\;(i=1,2,3).$ Presuming that each of the three rings undergoes a teeny virtual deformation, i.e., $\theta_{\xi_{i}}\left(s_{i}\right)=\theta_{i}\left(s_{i}\right)$ + $\xi_{i}\eta_{i}\left(s_{i}\right)$ (i=1,2,3), with $\xi_{1}$, $\xi_{2},$ and $\xi_{3}$ being different small positive parameters, we can express the first-order variation of W as follows:

(3) $$\begin{array}{ll} \delta W & =\sum_{i=1}^{3}\int_{0}^{\pi R_{i}}\left[{\left(\frac{\partial w_{i}}{\partial \theta_{i}^{\prime }}\right)}^{\prime }-\frac{\partial w_{i}}{\partial \theta_{i}}\right]\eta_{i}ds_{i}\\ & =\sum_{i=1}^{3}\int_{0}^{\pi R_{i}}\left(K_{i}\theta_{i}^{\prime \prime }-F_{x_{i}}\sin\theta_{i}+F_{y_{i}}\cos\theta_{i}\right)\;\eta_{i}ds_{i} \end{array}$$

Due to the arbitrariness of $\eta_{1}(s_{1})$, $\eta_{2}\left(s_{2}\right),$ and $\eta_{3}(s_{3})$, the equilibrium equations for the right half of the rings are obtained

(4) $$K_{i}\theta_{i}^{\prime \prime }\left(s_{i}\right)-F_{x_{i}}\sin\theta_{i}\left(s_{i}\right)+F_{y_{i}}\cos\theta_{i}\left(s_{i}\right)=0$$

where i=1,2,3. Additionally, the non-stretchable condition of the rings demands [38]

(5) $$x_{i}^{\prime }\left(s_{i}\right)=\cos\theta_{i}\left(s_{i}\right),y_{i}^{\prime }\left(s_{i}\right)=\sin\theta_{i}\left(s_{i}\right)$$

where i=1,2,3. Normally, the non-stretchable condition requires ${2\pi R}_{i}/t_{i}>100(i=1,2,3)$ so that the bending deformation of the rings dominate, and the stretchable deformation is negligible. It should be noted that the non-stretchable condition serves as a criterion for choosing sensor geometry parameters.

To obtain the numerical solution of the balance Equations (4) and (5), complementary conditions involving boundary and continuity are essential to achieve the consistent quantity between unknowns and equations. The variables ($\theta_{1}$, $\theta_{1}^{\prime }$, $x_{1}$, $y_{1}$, $\theta_{2}$, $\theta_{2}^{\prime }$, $x_{2}$, $y_{2}$, $\theta_{3}$, $\theta_{3}^{\prime }$, $x_{3}$, $y_{3}$) are continuous along certain flexible rings, with rings fulfilling the boundary conditions $\theta_{i}(0)=0$, $y_{i}$(0) = 0, $x_{i}$(0) = 0, $x_{i}$(π$R_{i}$) = 0, and $\theta_{i}$(π$R_{i}$) = π (i=1,2,3). By incorporating the aforementioned complementary conditions, the numerical solution of the equilibrium equations governing the rings’ deformation under the external compression force can be obtained (the function ‘bvp4c’ available in MATLAB was employed).

According to Equation (4), the correlation between the rings’ top displacement and the external compression force can be derived. According to Figure 1c, the original heights of the three flexible rings are denoted as 2$R_{1}$, 2$R_{2}$, and 2$R_{3}$ (dashed lines), respectively. Due to their nested configuration, the tops of these flexible rings exhibit identical downward displacement *∆y* under the loading of component compression forces $F_{y_{1}}$, $F_{y_{2}},$ and $F_{y_{3}}$. After numerically solving the equilibrium configuration of the compressed rings under the applied compression forces $F_{y_{1}}$, $F_{y_{2}},$ and $F_{y_{3}}$, and the rings’ top displacement *∆y* can be calculated as follows:

(6) $$\Delta y=2R_{i}-\int_{0}^{\pi R_{i}}\sin\theta_{i}\left(s_{i}\right)ds_{i}$$

where i=1,2,3.

The correlation between the rings’ top displacement and the applied component compression forces $F_{y_{1}}$, $F_{y_{2}},$ and $F_{y_{3}}$ can be derived using Equation (6), enabling the indirect measurement of the total compressive force $F_{compression}$ = $F_{y_{1}}+F_{y_{2}}+F_{y_{3}}$ by using the rings’ top displacement *∆y*. An analogous procedure can be employed to analyze the external tensile force loading.

## 3. FEM Simulation

**Figure 2:**
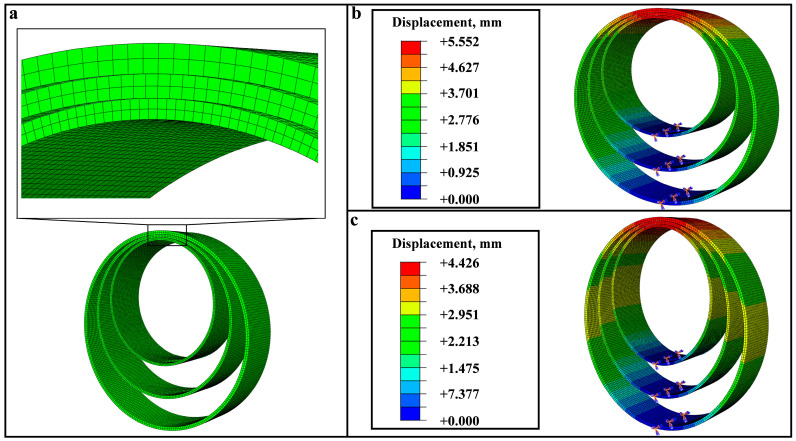
(**a**) FEM Meshes of the three rings model. Displacement nephogram of three flexible rings under (**b**) *F*_compression_ of 20 N and (**c**) *F*_tensile_ of 20 N.

To validate the effectiveness of the above analytical model, software ABAQUS was utilized to conduct Finite Element Method (FEM) simulations on the designed nested rings’ structure. A FEM model was developed for three nested flexible rings with the parameters: $R_{1}$ = 2.801 cm, $R_{2}$ = 3.599 cm, $R_{3}$ = 4.399 cm, $t_{1}$ = 1.13 mm, $t_{2}$ = 1.31 mm, $t_{3}$ = 1.63 mm, $b_{1}$ = 2.006 cm, $b_{2}$ = 2.007 cm, $b_{3}$ = 2.008 cm, density *ρ* = 1139.7 g/dm^3^, Poisson’s ratio *μ* = 0.1, and bending modulus *E* = 2050 MPa. The three rings’ top are bonded together by using the tie constraint so that the three rings can synchronously extend or contract when the loading force is loaded at the rings’ top, while the bottoms of the three rings remain fixed (fixing arrows in Figure 2b,c). The rings’ tops are constrained to move only in the *y* direction. We employed a static implicit procedure with an initial time step size of 0.01 to ensure the convergence. The quadric hexahedron elements (C3D20R) were used to mesh the FEM model with the reduction integral (Figure 2a). The mesh sizes of the three rings in the model are specified as 0.6 mm, 0.8 mm, and 1 mm, resulting in 246,516 nodes and 43,608 elements. A mesh convergence analysis was performed to validate the simulation’s accuracy.

**Figure 3:**
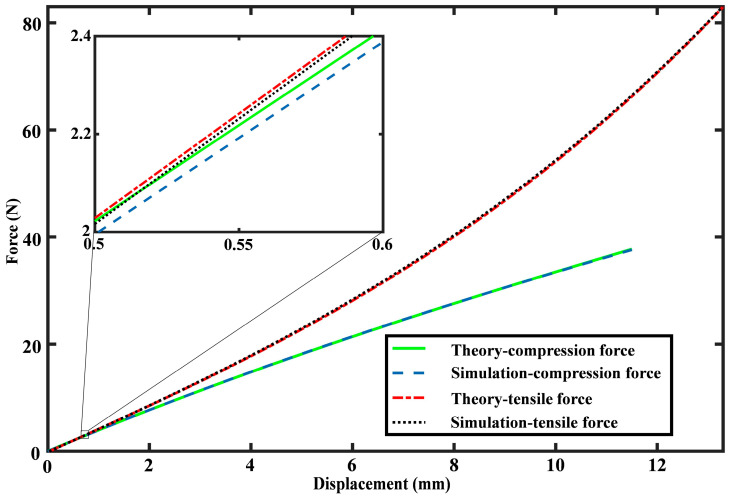
Force-displacement relationship comparison between simulation results and theoretical predictions.

According to the simulation results, displacement nephograms for three rings under the loading force is extracted, and the results of the 20 N loading are shown in Figure 2b (*F*_compression_) and Figure 2c (*F*_tensile_). According to nephograms, the rings’ top displacement ∆*y* is readily determined, and the force-displacement correlation is established by adjusting the loading force values during simulations. A comparison between the FEM simulation and theory is shown in Figure 3, where the theory is consistent with the FEM simulation. The maximal error between theory results and simulation within the measurement range is only 0.37%, proving that the proposed analytical model is precise.

## 4. Experimental Setup and Testing Results

### 4.1. Experimental Setup

**Figure 4:**
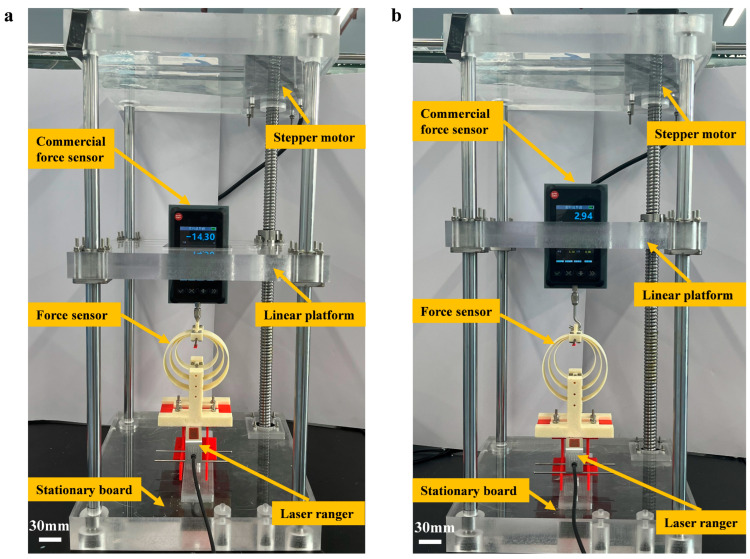
(**a**) Experiment setup for *F*_compression_. (**b**) Experiment setup for *F*_tensile_. Scale bars: 30 mm.

To assess the FS’s feasibility, experiments were conducted on the *F*_compression_ (Figure 4a) and the *F*_tensile_ (Figure 4b), respectively. The experiment setup includes the proposed FS, a customized linear platform, a commercial FS, and a laser ranger. The base of the FS is securely fastened to the stationary board so that the sensor remains fixed during tests. The commercial FS (DAYSENSOR DY920-B, Dayang Sensing Enterprise, Bengbu, China) (measurement error 0.1%) is fixed to the linear platform, allowing it to move vertically with the linear platform. A stepper motor (AnChuan 86HD2440, YASKAWA Electric Corporation, Kita-Kyushu, Japan) drives the vertical motion of the linear platform, and it is controlled by a stepper controller (AnChuan CA-01, YASKAWA Electric Corporation, Kita-Kyushu, Japan) and a driver (AnChuan CA-4060, YASKAWA Electric Corporation, Kita-Kyushu, Japan).

With the vertical movement of the linear platform, the loading part of the designed FS makes contact with the commercial FS, so either the *F*_compression_ or the *F*_tensile_ is applied to three flexible rings, and meanwhile, the commercial FS shows the applied force value on the screen. The loading part gravity is taken into account in the following force output calculation. A laser ranger (BOJKE BL-100NZ, Boyi Jingke Technology Co., Ltd., Shenzhen, China) (precision 75 μm and minimal reading 10 μm) is employed to gauge the three rings’ displacement. Three flexible rings are fabricated using Acrylonitrile Styrene Acrylate (ASA, Stratasys Ltd., Minneapolis, MI, USA) through a 3D printer (Stratasys F170, Stratasys Ltd., Minneapolis, MI, USA) (printing error 0.2 mm), and the bending modulus of ASA is 2050 MPa. A 0.4 mm nozzle width is employed to achieve good printing accuracy. The printing process is configured with a 2-layer shell for enhanced structural integrity. The infill density is set to 100% with a grid infill pattern. The layer thickness is set at 0.254 mm to balance the resolution and the printing time. The printing speed is set at 50 mm/s, and the temperature is maintained at 220 °C.

### 4.2. Force-Displacement Characteristic Experiment

**Figure 5:**
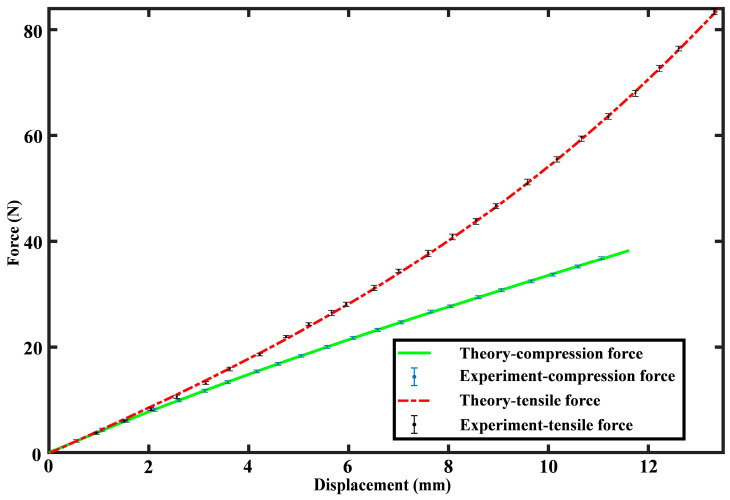
Force-displacement relationship comparison between experimental results and theoretical predictions.

By conducting *F*_compression_ and *F*_tensile_ experiments, we can derive the force-displacement characteristics of three flexible rings, which are then compared with theoretical predictions, as depicted in Figure 5. Owing to the 3D printer’s fabrication error, slight discrepancies in the ring geometry values exist between the initial design and the actual fabrication. The designed sizes of three elastic rings are ($R_{1}$, $t_{1}$, $b_{1}$) = (2.8 cm, 1 mm, 2 cm), ($R_{2}$, $t_{2}$, $b_{2}$) = (3.6 cm, 1.2 mm, 2 cm), and ($R_{3}$, $t_{3}$, $b_{3}$) = (4.4 cm, 1.5 mm, 2 cm). The dimensions of the printed rings are gauged using a vernier caliper, confirming that the non-stretchable condition is fulfilled, and the fabrication error of the printing process is considered. The actual manufactured sizes of three rings are ($R_{1}$, $t_{1}$, $b_{1}$, *E*) = (2.801 cm, 1.13 mm, 2.006 cm, 2050 MPa), ($R_{2}$, $t_{2}$, $b_{2}$, *E*) = (3.599 cm, 1.31 mm, 2.007 cm, 2050 MPa), and ($R_{3}$, $t_{3}$, $b_{3}$, *E*) = (4.399 cm, 1.63 mm, 2.008 cm, 2050 MPa), and the fabrication error of the 3D-printing can be observed. In the theoretical calculation of Figure 5, the actual manufactured sizes rather than the designed sizes of three elastic rings are used to eliminate the fabrication error. Three rings exhibited stable deformation under vertical loads in experiments, as demonstrated in a consecutive *F*_compression_ test (Supplementary Movie S1) and a consecutive *F*_tensile_ test (Supplementary Movie S2). Due to the nonlinear force-displacement characteristic, the proposed FS shows distinct measurement ranges in the *F*_compression_ test and the *F*_tensile_ test. Specifically, the measurement ranges of *F*_compression_ and *F*_tensile_ are 0–38 N and 0–83 N, while the measurement errors of *F*_compression_ and *F*_tensile_ are 0.69% and 0.77%, respectively. Besides, the resolution of the proposed sensor depends on the minimum output (0.01 mm) of the laser ranger, with a tensile force resolution of 0.042 N and a compression force resolution of 0.038 N.

### 4.3. Performance Test

**Figure 6:**
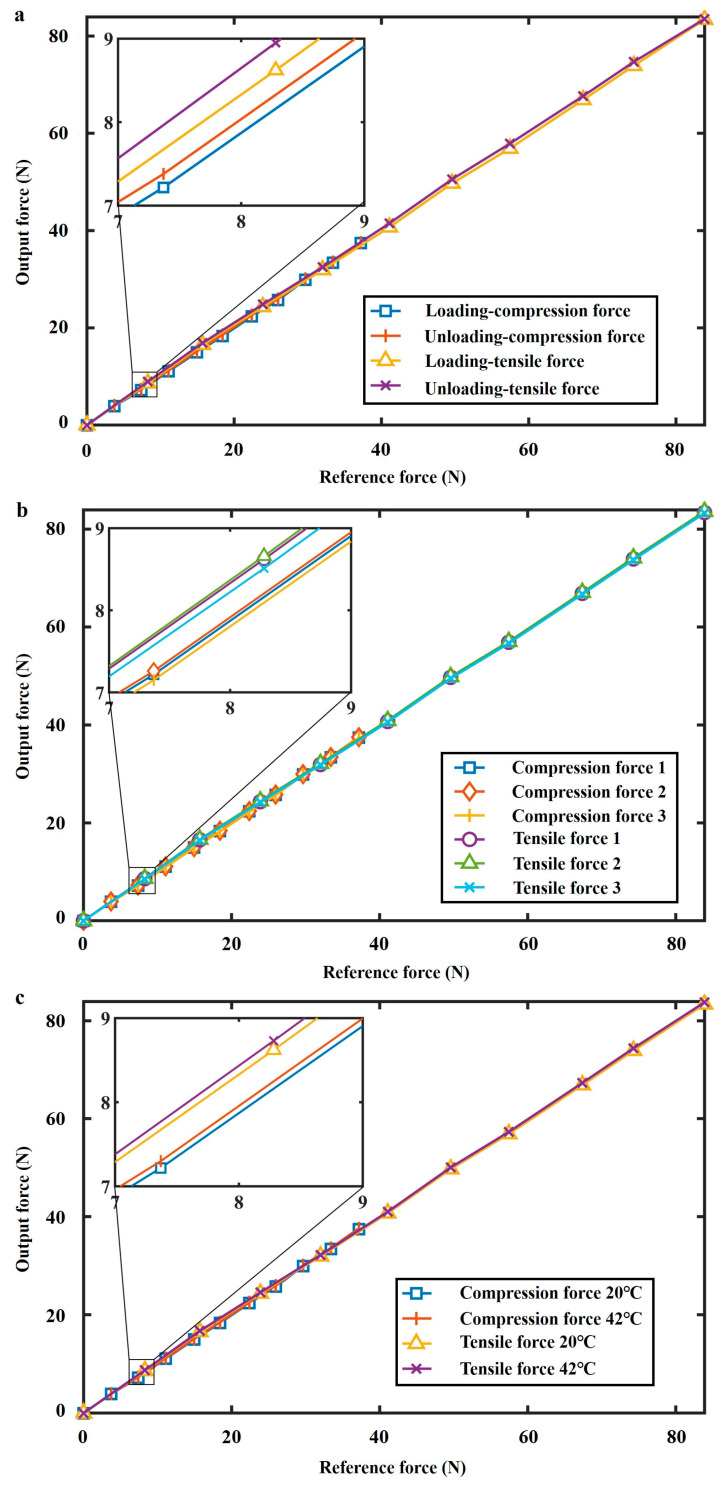
(**a**) Hysteresis test. (**b**) Repeatability test. (**c**) Temperature test. (42 °C and 20 °C).

To present the comprehensive performance of the designed FS, we tested the hysteresis (loading–unloading), straightness, repeatability, temperature, cyclic, and creep errors of the sensor, as shown in Table 1. For the hysteresis test in Figure 6a, the mean of three loading-unloading experiments is used to calculate the hysteresis error. The sensor’s hysteresis error is possibly attributed to its flexible structure. According to the loading test result of the hysteresis test in Figure 6a, the sensor’s straightness error is calculated. The sensor’s repeatability error is tested by loading the external force three times (Figure 6b).

**Figure 7:**
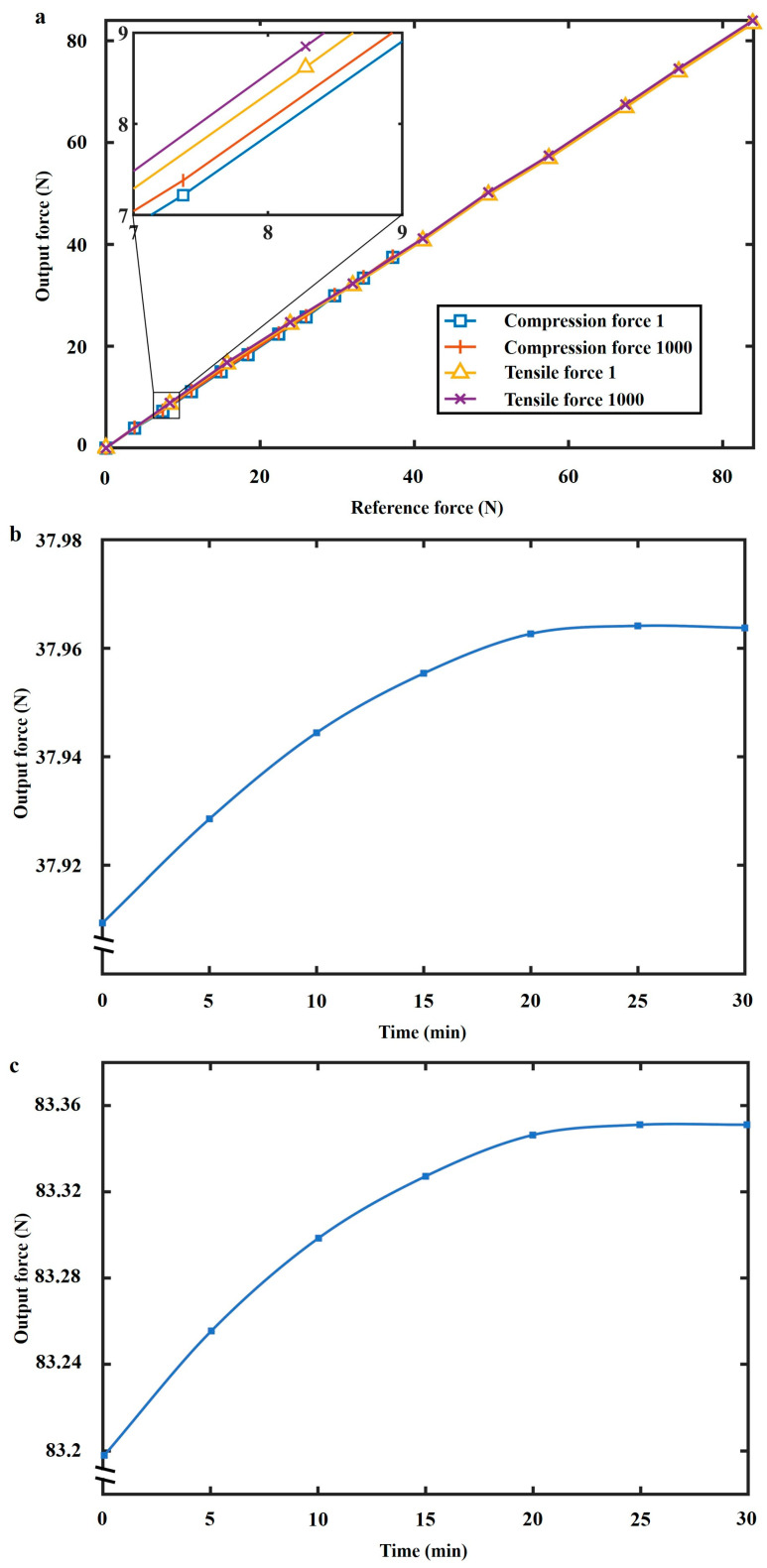
(**a**) Cyclic test. (**b**) *F*_compression_ creep test. (**c**) *F*_tensile_ creep test.

Temperature experiments (Figure 6c) and cyclic tests (Figure 7a) were conducted to show temperature characteristics and long lifespan. The temperature tests were carried out at 42 °C and 20 °C, respectively. The proposed sensor benefits from the stable temperature characteristics of the ASA material. We conducted the first loading test in the tensile force cyclic test and recorded the data as the line ‘Experiment-tensile force-1’ in Figure 7a. Then, 998 cycles of the loading tests were conducted without recording data. Until the 1000th cycle of the loading test, the testing data was recorded as the line ‘Experiment-tensile force-1000’ in Figure 7a. In the creep error test (Figure 7b,c), fixed rated loads were applied, and data was recorded at even time steps in half an hour.

## 5. Discussion

Based on the above analysis, the proposed FS shows good performance in terms of accuracy, weight, expenses, manufacture, and reliability. Table 2 provides a comparison between the proposed FS and previous counterparts. In contrast to FSs in the literature and commercial FSs (Burster 8524-series, Burster Enterprise, Gernsbach, Germany; Forsentek-F3F, Forsentek Enterprise, Shenzhen, China) using the strain gauge [30], the proposed FS offers the benefits of low expenses and reduced weight due to 3D-printing fabrication. The estimated total cost of the proposed FS is $28, and the detailed calculation process is as follows. The three elastic rings cost $5, which requires approximately 36.6 g of ASA material. The other parts cost $2.5, consuming approximately 150 g of PLA material. The bolts and iron rods used for assembly and fixing cost $1. The shell and indicator controller are expected to be $4.5. Additionally, we have used the lowest price (including the second-hand price) of the laser ranger (BOJKE BL-100NZ, Boyi Jingke Technology Co., Ltd., Shenzhen, China) available in the market, which costs $15.

The measuring principle of the proposed FS is valid for both the macro-scale and the micro-scale when the geometry parameters of the ring satisfy 2π*R*/*t* > 100 or *t* < π*R*/50. This design not only holds the potential for constructing the micro-scale FS but also facilitates applications with small size and weight requirements. Taking the micro-sized FS as an example, a ring with *t* of 0.4 mm should satisfy *R* > 6.37 mm. For the Stratasys F170 printer, it is feasible to print micro-sized elastic rings with ($R_{1}$, $t_{1}$, $b_{1}$, *E*) = (0.65 cm, 0.4 mm, 0.5 cm, 2.05 GPa), ($R_{2}$, $t_{2}$, $b_{2}$, *E*) = (0.8 cm, 0.5 mm, 0.5 cm, 2.05 GPa), and ($R_{3}$, $t_{3}$, $b_{3}$, *E*) = (0.96 cm, 0.6 mm, 0.5 cm, 2.05 GPa), which can be integrated with the micro-sized displacement sensor, e.g., micro-fiber optic displacement sensor [30,32], to realize micro-sized FSs. However, after miniaturization, the measurement range will be reduced accordingly, and the measurement range for the above micro-sized rings is 20.78 N. Micro-scale FS has good potential for application in medical equipment. For example, Asghar and Tang et al. [30,32] combined the prismatic-tip optical fiber sensing with the planar spring to develop a micro-sized FS, which can accurately measure the pulse waveform of the radial artery and be used in the diagnosis and examination of cardiovascular diseases.

By utilizing 3D additive manufacturing technologies, the proposed FS is efficiently manufactured using materials possessing the required characteristics and proper geometries, thereby establishing an economical force-sensing solution tailored to diverse application needs. For example, in the automobile industry, a large measurement range is usually required for mechanical testing of various automotive components, such as chassis parts and structural elements. The proposed FS with geometry parameters in Section 4.2 would achieve a measurement range of 2 kN for three rings with *E* = 49.4 GPa, and the larger measurement range can be achieved using larger rings or using material with a larger elastic modulus. In robotics applications, a small size and a large measurement range are usually required for single-axis force testing in a robot arm or hand. For example, the proposed FS would achieve a small size of 2.5 cm and a measurement range of 190 N for three rings with ($R_{1}$, $t_{1}$, $b_{1}$, *E*) = (0.75 cm, 0.4 mm, 0.6 cm, 20.5 GPa), ($R_{2}$, $t_{2}$, $b_{2}$, *E*) = (1 cm, 0.6 mm, 0.6 cm, 20.5 GPa), and ($R_{3}$, $t_{3}$, $b_{3}$, *E*) = (1.25 cm, 0.7 mm, 0.6 cm, 20.5 GPa).

Traditional 3D-printed FSs often meet substantial errors [30,31,32,33] attributed to nonlinear force responses, and their complicated structures pose challenges in developing nonlinear theories describing these behaviors. Owing to the straightforward measurement principle and the established analytical model, this FS exhibits smaller measurement errors compared with previous 3D-printed FSs. In contrast with our previous work [5], the proposed FS significantly reduces the geometry parameters and has a measurement range close to that of the previous work, although the measurement error slightly increases due to the influence of additional structures.

Selecting materials with a larger modulus for fabricating the rings enhances the potential to accomplish compact dimensions and larger ranges and meanwhile keeps the good accuracy and low fabrication expenses. This study concentrates on assessing the effectiveness of the force measurement principle, and thus, a small modulus is adopted. Further enhancing the measurement accuracy of the proposed sensor entails refining the proposed analytical model. For instance, incorporating stretchable deformations into the analytical model is anticipated to enhance sensor accuracy. Future research will focus on studying these problems, including measurement range improvement, system integration, and accuracy improvement.

## 6. Conclusions

This paper proposes a novel FS employing three 3D-printed nested flexible rings, and the corresponding sensing principle is elaborated. An analytical model on the basis of the minimal energy method is developed to elucidate the force-displacement correlation with nonlinearity. Both the FEM simulation and experiment test prove the feasibility of the proposed measurement principle and show a measurement error of less than 0.77%. As a primary study of a novel force measurement method, this work concentrates on the validation of the measurement scheme. Future research will concentrate on improvement in measurement range, system integration, and accuracy. Overall, the proposed FS not only enhances the precision of 3D-printed FSs but also facilitates the decrease in cost and weight for high-accuracy FSs.

## Figures and Tables

**Table 1:** Comprehensive performance of the FS.

Force	Hysteresis Error	Straightness Error	Repeatability Error	Temperature Error	Cyclic Error	Creep Error
Compression Force	0.71%	0.69%	0.42%	0.48%	0.62%	0.13%
Tensile Force	1.18%	0.77%	0.46%	0.56%	0.73%	0.19%

**Table 2:** Comparison between the proposed FS and reported FSs.

FS	Error	Range	Dimension	Applications	Mass	Cost
Tandem FS [19]	1.54%	0–819 N	Length 7 cm Diameter 3.2 cm	Robotics and perceptual information	\	\
Axis-Symmetrical Force Transducers [20]	3.0%	0–50 kN	Length 19 cm Breadth 5 cm Height 19 cm	Automation industries and verification of material testing machines	\	\
MEMS FS [21]	1.0%	0.5–5 N	Length 1.1 cm Breadth 0.12 cm Thickness 0.06 cm	Automotive industry	\	\
FS with novel strain gauge [22]	1.78%	0–800 N	Length 1.75 cm Breadth 0.8 cm Height 0.8 cm	Intelligent automation of robot	\	\
Novel six-axis FS [23]	2.0%	0–800 N	Length 1.75 cm Breadth 0.8 cm Height 0.8 cm	Robotics	\	\
Mechanical decoupling FS [24]	5.2%	0–800 N	Length 3.7 cm Diameter 9.2 cm	Biomechanics and sports medicine	\	\
Weight sensing device [27]	5.0%	0–500 kN	Length 40 cm Breadth 12.5 cm Height 5 cm	Detection of heavy objects	\	\
Flexible FS based on the hall effect [28]	6.9%	0–40 N	Diameter 6 cm Height 1.1 cm	Tactile and healthcare applications	\	\
Novel sensor for robot applications [29]	1.18%	0–50 N	Diameter 2.5 cm Height 1.9 cm	Robot applications	\	\
Planar force and torque sensors [30]	0.29%	0–0.1 kN	Diameter 7.5 cm Height 1.2 cm	Robotic systems	\	\
3D-printed FS utilizing elastic elements arranged orthogonally [31]	2.18%	0–2.8 N	Diameter 7 cm Height 0.4 cm	Automated manipulation and advanced manufacturing	\	\
3D-printed micro-FS [32]	1.14%	0–0.12 N	Length 3.5 cm Breadth 1 cm Height 0.3 cm	Microscale integration, healthcare implementations and fine-tuning of microdevices	\	\
3D-printed FS utilizing optical fiber grating [33]	0.98%	0.9–2.7 N	Length 5.7 cm Diameter 3 cm	Analysis of pulse patterns	\	\
Forsentek-F3F (Forsentek Enterprise)	0.3%	0–2 kN	Length 12 cm Breadth 12 cm Height 3 cm	Automated systems, force simulation platform, and industrial testing	3.2 kg	$1236
DAYSENSOR DY920-B (Dayang Sensing Enterprise)	0.1%	0–100 N	Length 13.2 cm Breadth 7.6 cm Height 2 cm	Industrial production and automation	0.5 kg	$123
Burster 8524-series (Burster Enterprise)	0.1%	0–2 kN	Height 1.6 cm Diameter 5.45 cm	Machinery industry and batch weighing apparatus	0.57 kg	$751
This work	0.77%	0–83 N	Length 9.8 cm Breadth 7 cm Height 8.8 cm	Robotics, automotive industry, and iatrical equipment	0.3 kg	$28

## Data Availability

The data that support the findings of this study are available from the corresponding author, F.H., upon reasonable request.

## Supplementary Materials

The following supporting information can be downloaded at: https://www.mdpi.com/article/10.3390/s24165441/s1, Movie S1: Continuous compression force test, Movie S2: Continuous tensile force test.
